# Functional analysis of XRCC4 mutations in reported microcephaly and growth defect patients in terms of radiosensitivity

**DOI:** 10.1093/jrr/rrab016

**Published:** 2021-04-12

**Authors:** Anie Day D C Asa, Rujira Wanotayan, Mukesh Kumar Sharma, Kaima Tsukada, Mikio Shimada, Yoshihisa Matsumoto

**Affiliations:** Laboratory for Advanced Nuclear Energy, Institute of Innovative Research, Tokyo Institute of Technology, Tokyo 152-8550, Japan; Laboratory for Advanced Nuclear Energy, Institute of Innovative Research, Tokyo Institute of Technology, Tokyo 152-8550, Japan; Department of Radiological Technology, Mahidol University, Nakhon Pathom 73170, Thailand; Laboratory for Advanced Nuclear Energy, Institute of Innovative Research, Tokyo Institute of Technology, Tokyo 152-8550, Japan; Department of Zoology, SPC Government College, Ajmer-305001, Rajasthan, India; Laboratory for Advanced Nuclear Energy, Institute of Innovative Research, Tokyo Institute of Technology, Tokyo 152-8550, Japan; Laboratory for Advanced Nuclear Energy, Institute of Innovative Research, Tokyo Institute of Technology, Tokyo 152-8550, Japan; Laboratory for Advanced Nuclear Energy, Institute of Innovative Research, Tokyo Institute of Technology, Tokyo 152-8550, Japan

**Keywords:** DNA double-strand break (DSB) repair, non-homologous end joining, XRCC4, microcephaly, growth defect, radiosensitivity

## Abstract

Non-homologous end joining is one of the main pathways for DNA double-strand break (DSB) repair and is also implicated in V(D)J recombination in immune system. Therefore, mutations in non-homologous end-joining (NHEJ) proteins were found to be associated with immunodeficiency in human as well as in model animals. Several human patients with mutations in XRCC4 were reported to exhibit microcephaly and growth defects, but unexpectedly showed normal immune function. Here, to evaluate the functionality of these disease-associated mutations of XRCC4 in terms of radiosensitivity, we generated stable transfectants expressing these mutants in XRCC4-deficient murine M10 cells and measured their radiosensitivity by colony formation assay. V83_S105del, R225X and D254Mfs^*^68 were expressed at a similar level to wild-type XRCC4, while W43R, R161Q and R275X were expressed at even higher level than wild-type XRCC4. The expression levels of DNA ligase IV in the transfectants with these mutants were comparable to that in the wild-type XRCC4 transfectant. The V83S_S105del transfectant and, to a lesser extent, D254Mfs^*^68 transfectant, showed substantially increased radiosensitivity compared to the wild-type XRCC4 transfectant. The W43R, R161Q, R225X and R275X transfectants showed a slight but statistically significant increase in radiosensitivity compared to the wild-type XRCC4 transfectant. When expressed as fusion proteins with Green fluorescent protein (GFP), R225X, R275X and D254Mfs^*^68 localized to the cytoplasm, whereas other mutants localized to the nucleus. These results collectively indicated that the defects of XRCC4 in patients might be mainly due to insufficiency in protein quantity and impaired functionality, underscoring the importance of XRCC4’s DSB repair function in normal development.

## INTRODUCTION

DNA double-strand break (DSB) is the most detrimental damage to DNA and occur upon genotoxic stresses such as ionizing radiation and replication errors, or as intermediates in recombination. Eukaryotic cells have two major pathways to repair DNA DSBs: homologous recombination (HR) and non-homologous end-joining (NHEJ) [1]. Although HR is considered more accurate than NHEJ, HR requires sister chromatid as the template; therefore, it is limited to late S and G2 phases [1]. NHEJ is predominant in mammalian cells and plays an essential role in V(D)J recombination, which generates diversity of immunoglobulins and T cell receptors in lymphogenesis [1]. Core factors for NHEJ include heterodimeric Ku protein (Ku86 and Ku70), DNA-dependent protein kinase catalytic subunit (DNA-PKcs), X-ray cross complementing 4 (XRCC4), XRCC4-like factor (XLF, also known as Cernunnos), Paralog of XRCC4 and XLF (PAXX) and DNA ligase IV (LIG4) [1]. Animal models or human individuals lacking one of these factors exhibit cellular hypersensitivity to radiation and defective V(D)J recombination, leading to severe combined immunodeficiency, which is deficient for both of B cells and T cells [1].

**Fig. 1.**
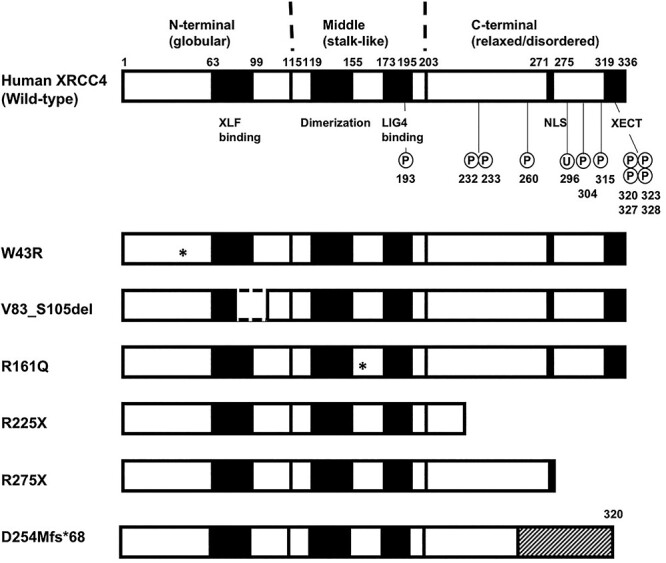
Schematic diagram of the structure of human XRCC4 and disease-associated mutants. Phosphorylation sites (P) and ubiquitylation sites (U) are indicated in wild-type XRCC4. NLS: nuclear localization signal, XECT: XRCC4 extremely C-terminal region. Asterisks in W43R and R161Q indicate the positions of the mutations. Hatched region in D254Mfs^*^68 shows a completely changed amino acid sequence due to the frameshift mutation.

XRCC4 was initially found as the gene which could restore normal V(D)J recombination and DNA DSB repair ability to Chinese hamster ovary-derived XR-1 cells [2]. XRCC4 consists of a globular N-terminal domain (amino acids 1–115), a stalk-like middle domain (amino acids 116–203), and a relaxed/disordered C-terminal region (amino acids 204–336, note that there is an alternatively spliced form consisting of 334 amino acids, in which Asn298-Ser299-Arg300 are replaced with lysine) [3, 4] (Fig. 1). XRCC4 forms a homodimer or a homotetramer through its coiled-coil domain [3, 4]. Although XRCC4 did not show any similarity to known proteins at the time of its identification, four XRCC4 paralogs are now identified in the human genome: XLF [5, 6], PAXX [7–9], SAS6 [7] and CCDC61 [10]. All these paralogs share N-terminal globular structures and stalk-like structures, which mediate homodimer formation. Whereas XRCC4, XLF and PAXX are essential for NHEJ [2, 5–9], SAS6 and CCDC61 are involved in the regulation of centrosome [7, 10]. XRCC4 has no known enzymatic activity but interacts with LIG4 [11, 12]. XRCC4 was also shown to be required for the stability and nuclear localization of LIG4 [13–15]. It was suggested that XRCC4 interacts with XLF through its N-terminal globular region to form a filament, which may align or bridge two DNA ends [16–19]. Moreover, XRCC4 was shown to interact with Polynucleotide kinase/phosphatase (PNKP) [20, 21], Aprataxin (APTX) [22] and PNKP- and APTX-like FHA protein (PALF, also known as APLF) [23–25], which may be mediated between phosphorylated Ser232 and/or Thr233, by Casein kinase II, of XRCC4 and Forkhead-associated domains of these proteins [26]. Finally, XRCC4 was shown to undergo phosphorylation by DNA-PK *in vitro* and in cellulo [12, 27–29]. Major phosphorylation sites *in vitro* and in cellulo, i.e. Ser260 and Ser320, were in the C-terminal region, but additional phosphorylation sites, i.e. Ser193, Ser304, Ser315, Thr323, Ser327 and Ser328, were also suggested [30–33]. Although the role of each phosphorylation site was not sufficiently clarified, mutating all of above phosphorylation sites together resulted in a significant reduction in XRCC4 function [34]. It was also noted that approximately 20 amino acids located at the extremely C-terminal (XECT) region of human XRCC4, containing four of the above potential phosphorylation sites, are highly conserved among vertebrate species and Asn326 therein was shown to be essential for XRCC4 function [35]. XRCC4 undergoes M-phase specific phosphorylation by Cyclin-dependent kinase and/or Polo-like kinase 1 (PLK1), which was suggested to be in the C-terminal region [36]. Additionally, Lys296 was shown to undergo K63-linked polyubiquitylation by F-box and WD repeat domain-containing 7 (FBXW7) ubiquitin ligase E3, which was triggered by DNA-PK [37]. Thus, the C-terminal region may regulate XRCC4 function through post-translational modifications and protein–protein interactions.

Studies in mice showed that the deficiency in XRCC4 as well as LIG4 caused late embryonic lethality, associated with defective lymphogenesis and neurogenesis [38, 39]. In 2014–2015, several human patients exhibiting microcephaly and/or growth defects, were found to harbor mutations in XRCC4 [40–45] (Table 1 and extended form in Table S1). Unexpectedly, however, none of these patients were reported to exhibit overt immunological disorders [40–47]. This prompted us to study the impact of these disease-associated XRCC4 mutations on its function in DNA repair. We generated XRCC4 mutants mimicking these mutations and evaluated their functionality in terms of radiosensitivity measured by colony formation assay, along with protein expression and nuclear localizing ability.

**Table 1:** Profile of Patients associated with XRCC4 Mutations.

Patient Gender Country	Change in nucleotide sequence	Change in amino acid sequence	Clinical features		Reference
			Microcephaly (OFC)	Short statue (Length/height)	
P1 Female Saudi Arabia	c.T127C (Homozygous)	p.W43R	Y (4y:-8.3SD)	Y (4y:-7.1SD)	[40]
P2 Male Saudi Arabia	c.T127C (Homozygous)	p.W43R	Y (Birth:-4.87SD; 3y1m:-8.3SD)	Y (Birth:-2.52SD; 3y1m:-4.7SD)	[41]
P3 Male Morocco	c.C481T c.C673T	p.R161X p.R225X	Y (Birth:-4.57SD; 2y9m:-8.3SD)	Y (Birth:-6.32SD; 2y9m:-5.7SD)	[41]
P4–1^#^ Male Italy	c.C25del c.C823T	p.H9Tfs^^*^^8 p.R275X	Y (Birth:-2.9SD 8y4m:-5.6SD)	Y (Birth:-4.49SD 8y4m:-2.4SD)	[41]
P4–2^#^ Male Italy	c.C25del c.C823T	p.H9Tfs^^*^^8 p.R275X	Y (Birth:-1.83SD; 4y:-8.0SD)	Y (Birth:-5.38SD; 4y:-4.5SD)	[41]
P5 Male France	c.C25del c.C823T	p.H9Tfs^^*^^8 p.R275X	Y (Birth:-4.28SD; 9y:-5.8SD)	Y (Birth:-2.71SD; 9y, −1.8SD^^*^^)	[41]
P6 Male United Kingdom	c.C25del c.G-10-1 T	p.H9Tfs^^*^^8 splicing defect	Y (Birth:-2.9SD; 5 m, −8.9SD)	Y (Birth:-6.56SD; 5 m, −7.2SD)	[41]
P7–1^#^ Male Italy	c.C673T (Homozygous)	p.R225X	NR	Y	[42]
P7–2^#^ Male Italy	c.C673T (Homozygous)	p.R225X	NR	Y	[42]
P8–1^#^ Male Chile	*c.T246G, c.247_315del* (Homozygous)	p.D82E, p.V83-S105del	Y (39.9y:-3.3SD)	Y (Birth:-2.8SD; 39.9y:-6.8SD)	[43]
P8–2^#^ Female Chile	*c.T246G, c.247_315del* (Homozygous)	p.D82E, p.V83-S105del	Y (36y:-2.9SD)	Y (Birth:-2.3SD; 36y:-4.0SD)	[43]
P9–1^#^ Male Turkey	c.G482A (Homozygous)	p.R161Q	Y (Birth:<-3SD; 14y:-7.5SD)	Y (Birth:-2 SD; 14y:-5SD)	[44]
P9–2^#^ Male Turkey	c.G482A (Homozygous)	p.R161Q	Y (Birth:<-3SD; 10.5y:-6.5SD)	Y (Birth:NR; 10.5y:-5SD)	[44]
P9–3^#^ Male Turkey	c.G482A (Homozygous)	p.R161Q	Y (Birth:<-3SD; 6 m:-4.5SD)	Y (Birth:NR; 6 m:-3SD)	[44]
P10 Female Switzerland	c.C25del c.C823T	p.H9Tfs^^*^^8 p.R275X	Y (Birth:-3.7SD; 14y10m:-5.0SD)	Y (Birth:-2.8SD; 14y10m:-2.6SD)	[44]
P11 Female United Kingdom	c.C673T c.G760del	p.R225X p.D254Mfs^^*^^68	Y	Y	[45]

^#^: siblings.

^^*^^Growth hormone-treated.

Abbreviations: Y, yes; NR, not reported; y, year; m, month; SD, standard deviation; OFC, occipitofrontal circumference.

## MATERIALS AND METHODS

### Plasmid construction and mutagenesis

Human XRCC4 cDNA was obtained from polymerase chain reaction (PCR) of the cDNA pool of human T-cell leukemia MOLT-4 cells using the primers X4-FL-F, CCG AAT TCC *ATG* GAG AGA AAA ATA AGC AGA ATC (the *Eco*RI restriction sequence is underlined and the initiation codon is italicized) and X4-FL-R, TCT AGA TC*T**TA*C TCG AGA ATC TCA TCA AAG AGG TC (the *Bam*HI and *Xho*I restriction sequences are underlined and the stop codon is italicized). The PCR product was digested by *Eco*RI and *Bam*HI and was integrated into p3xFLAG-CMV-10 (Sigma-Aldrich; St. Louis, MO, USA) to express XRCC4 protein with triple tandem FLAG epitope [48]. Because of the insertion of *Xho*I restriction site before the stop codon, leucine and glutamic acid residues were added at the C-terminus of the full-length proteins. To express XRCC4 as a fusion protein with Green fluorescent protein (GFP), the cDNA of XRCC4 was excised from p3xFLAG-CMV-10 vector using *Eco*RI and *Bam*HI and was inserted into pEGFP-C1 vector (Clontech, Mountain View, CA, USA) [15, 35]. The maps of p3xFLAG-CMV-10-XRCC4 and pEGFP-C1-XRCC4 plasmids are shown in suppmentary data (Figs Fig. S1 and Fig. S2, respectively).

Point, frameshift and deletion mutations were introduced using PrimeSTAR Mutagenesis Kit (Takara Bio; Otsu, Shiga, Japan). Sequences of PCR Primers for mutagenesis were as follows (mutated nucleotides are underlined and hyphen (−) indicates deletion): W43R-F, TCA GCA CGG ACT GGG ACA GTT TCT GAA; W43R-R, CCC AGT CCG TGC TGA ATG ACC ATC AGT; R161Q-F, CAA GGA CAA TTT GAA AAA TGT GTG AGT; R161Q-R, TTC AAA TTG TCC TTG AAC ATC ATT CCA; V83_S105del-F, GCT GAT TTC AGA CTT GGT TCC TTC; V83_S105del-R, TCT GAA ATC AGC TGG TCC TGC TCC; R225X-F GCT GAC TGA GAT CCA GTC TAT GAT GAG; R225X-R, TGG ATC TCA GTC AGC AGT CAT TTC AGA; R275X-F, AGA CAG TGA ATG CAA AGA AAT CTT GGG; R275X-R, TTG CAT TCA CTG TCT CCT TTT TCT ACT; D254Mfs^*^68-F, AGT AAA -AT GAT TCC ATT ATT TCA AGT; D254Mfs^*^68-R, GGA ATC AT- TTT ACT TAC AGC AGC TGA. Note that, the codons beyond the premature stop codon were retained in R225X and R275X. The entire XRCC4 open reading frames of the constructs were then verified to be the correct sequence by sequencing for all the constructs.

### Cell culture

M10 cell line, a derivative of murine leukemia cell line L5178Y, harboring a nonsense mutation in XRCC4 gene (c.A370T, p.R124X) [49, 50], was obtained from RIKEN cell bank (Tsukuba, Ibaraki, Japan; Code RCB0136) with permission of Dr. Koki Sato (National Institute of Radiological Sciences and Kinki University). M10 and its transfectants were cultured in RPMI1640 medium supplemented with 10% v/v fetal bovine serum (FBS) and 100 units/mL penicillin and 100 μg/mL streptomycin at 37 °C in humidified atmosphere containing 5% CO_2_. Human osteosarcoma cell line U2OS was obtained from American Type Culture Collections and was cultured in Dulbecco’s modified Eagle’s medium containing 4.5 g/L glucose supplemented with 10% v/v FBS, 100 units/mL penicillin and 100 μg/mL streptomycin at 37 °C in humidified atmosphere containing 5% CO_2_. FBS was purchased from HyClone (Logan, UT, USA). Medium and other reagents were purchased from Nacalai Tesque (Kyoto, Japan).

### Transfection

For M10 cells, plasmids were transfected by electroporation using Neon Transfection system (Invitrogen; Carlsbad, CA, USA). About 10^6^ M10 cells were transfected with 1 µg of plasmid DNA and allowed to grow in RPMI1640 medium with 10% v/v FBS in 48 hours. Transfected cells were then plated in RPMI1640 medium supplemented with 15% v/v FBS, 100 units/mL penicillin, 100 μg/mL streptomycin, 0.8 mg/mL G418 and 0.2% agarose (Becton, Dickinson and Company; Franklin Lakes, NJ, USA). Two weeks after plating, visible colonies were picked up and expanded to obtain the stably transfected clones of the different mutated XRCC4 constructs.

For U2OS cells, plasmids were transfected using Lipofectamine 2000 Reagent (Invitrogen). Subcellular localization of GFP-tagged XRCC4 was observed two days after cDNA transfection using inverted fluorescence microscope IX71 (Olympus; Tokyo, Japan).

### Irradiation and colony formation assay

Cells were irradiated using ^60^Co γ-source (222 TBq as of February 2010). Dose rate was measured using ionizing chamber-type exposure dosimeter C-110 (Oyo-Giken, Tokyo, Japan). The time to obtain the desired dose was calculated, considering the time required for ascending and descending the radiation source.

The protocol to measure the radiosensitivity of M10 cells by colony formation assay in soft agarose was previously described [16–17]. Briefly, cells suspended in complete medium were irradiated and then diluted to obtain appropriate number of cells. Cells were plated in triplicate on 6-cm dishes containing 0.25% w/v agarose in RPMI1640 solution with 15% v/v FBS, 100 units/mL penicillin and 100 μg/mL streptomycin per sample. Cells were allowed to grow for 12–14 days at 37 °C in humidified atmosphere containing 5% CO_2_. Plating efficiency (PE) was calculated as the number of colonies divided by the number of cells plated. Surviving fraction (SF) was calculated as PE of irradiated cells divided by PE of unirradiated control. Statistical significance of difference was tested by two-way factorial analysis of variance, setting cells and doses as the factors.

### Western blotting

Anti-XRCC4 rabbit polyclonal antibody [47], anti-FLAG mouse monoclonal antibody (clone M2; F3165; Sigma-Aldrich; St. Louis, MO, USA), anti-Glyceraldehyde-3-Phosphate Dehydrogenase (GAPDH) mouse monoclonal antibody (clone 6C5; MAB374;) and anti-LIG4 guinea pig polyclonal antibody (gifted by Prof. Miki Shinohara, Kinki University) [36] were used as the primary antibody at 1/1000 to 1/5000 dilution. As the secondary antibody, horseradish peroxidase-conjugated anti-rabbit immunoglobulins swine polyclonal antibody (P0399; Dako; Glostrup, Denmark), anti-mouse immunoglobulins goat polyclonal antibody (P0447; Dako) or anti-guinea pig immunoglobulins rabbit polyclonal antibody (P0141; Dako) was used at 1/1000 to 1/3000 dilution. The immunocomplexes were developed using WesternSure Chemiluminescent Western Blot Reagent (LI-COR; Lincoln, NE, USA) and the images were captured by C-Digit Blot Scanner (LI-COR). Protein Ladder One Plus, Triple-color (Nacalai Tesque) was used as the molecular weight standard. Other procedures of western blotting followed earlier publications [31, 32].

## RESULTS

### Stable transfectants of M10 expressing XRCC4 mimicking mutations in patients

**Fig. 2.**
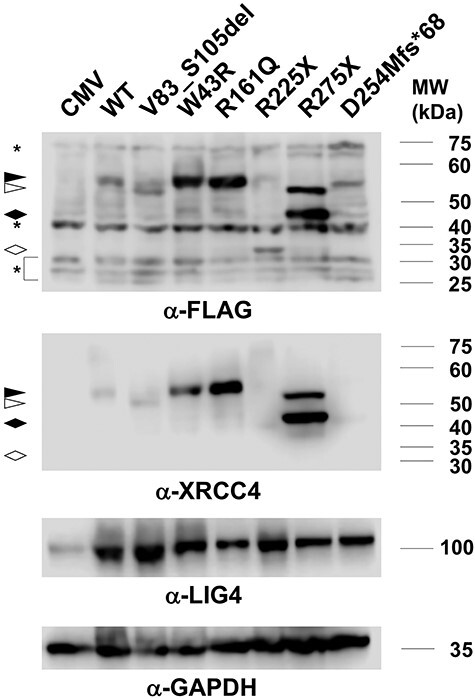
Expression levels of XRCC4 in M10-transfectants with wild-type and disease-associated mutants of XRCC4. Extracts from the transfectants (20 μg of proteins) were loaded and analyzed by western blotting using anti-FLAG, anti-XRCC4, anti-LIG4 or anti-GAPDH as the loading control. In anti-FLAG and anti-XRCC4 blots, XRCC4 bands are indicated as follows. Filled triangles: WT, W43R, R161Q and D254Mfs^*^68, open triangles: V83_S105del and R275X (upper), filled diamonds: R275X (lower), open diamonds: R225X. The stars in anti-FLAG blot indicate cross-reactive bands.

Disease-associated XRCC4 mutations reported in literatures are shown in Table 1 with their locations in XRCC4 structure in Fig. 1. We generated XRCC4 mutants mimicking six of the mutations found in patients with microcephaly and growth defect. We did not generate H9Tfs^*^8, as it lacks almost all of the XRCC4 structure, and also R161X, as it lacks the LIG4-interacting region. cDNA vectors to express these proteins with 3XFLAG tag were introduced into M10 cell, which is deficient for endogenous XRCC4, and stable transfectants were obtained. The expression of mutant XRCC4 was examined by Western blotting using anti-XRCC4 and anti-FLAG antibodies. Results using anti-XRCC4 and anti-FLAG antibodies were mostly consistent, except for R225X and D254Mfs^*^68, which were not detected by anti-XRCC4. V83_S105del, R225X and D254Mfs^*^68 was expressed at a similar level to wild-type XRCC4 and W43R, R161Q and R275X were expressed at even higher level than wild-type XRCC4 (Fig. 2). While the mobilities of W43R, R161Q and D254Mfs^*^68 were similar to that of wild-type, V83_S105del, R225X and R275X exhibited higher mobilities as expected from their molecular masses. R275X showed two bands of 50–55 kDa and 40–45 kDa in apparent molecular masses, respectively, the latter being more intense than the former. The upper band exhibited similar mobility to V83_S105del, and appeared considerably larger than the expected size of R275X. It was reported that 3XFLAG-tagged XRCC4(1–265), which was produced by Caspase-3, exhibited the apparent molecular mass slightly larger than 37 kDa [51]. Since the difference in the molecular mass of R275X and XRCC4(1–265) is 1.1 kDa, the lower band would be more likely to be R275X. The identity of the upper band in R275X is currently unclear.

Since XRCC4 was shown to be required for the stability of LIG4 [13], the expression of LIG4 in these transfectants was also examined (Fig. 2). The expression of LIG4 in the mutant XRCC4 transfectants was comparable to that in the wild-type XRCC4 transfectant, although it was substantially reduced in the control vector transfectant.

### Functionality of XRCC4 mimicking mutations of patients in terms of radiosensitivity

**Fig. 3.**
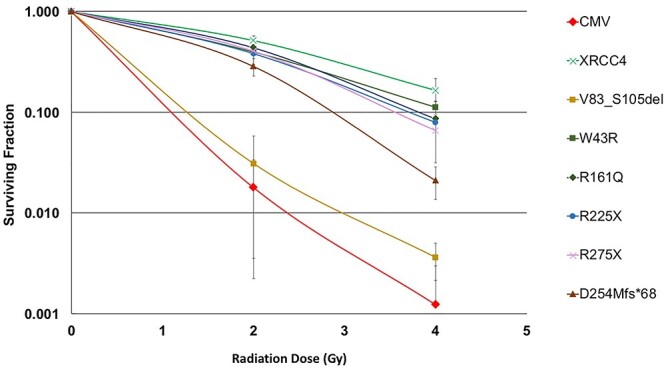
Radiosensitivity of M10-transfectants with wild-type and disease-associated mutants of XRCC4 measured by colony formation assay. Symbols and error bars indicate the mean and the standard deviation in 3 to 4 repeated experiments, respectively.

The radiosensitivity of M10 transfectants with XRCC4 mutants was assessed by colony formation assay after 2 and 4 Gy of γ-ray irradiation using cobalt 60 source (Fig. 3). Statistical significance of the difference between the transfectants was tested by two-way factorial analysis of variance. V83_S105del transfectant showed the highest radiosensitivity (p = 2.1E-8 with wild-type), which was close to that of the control CMV vector transfectant (p = 1.1E-8 with wild-type). Although S.F. of V83_S105del transfectant after 2 and 4 Gy irradiation looked somewhat higher than that of CMV transfectant, the difference was not statistically significant (p = 0.42). D254Mfs^*^68 transfectant showed substantially increased radiosensitivity compared to wild-type XRCC4 transfectant (p = 5.6E-4) but was still less sensitive than CMV transfectant (p = 7.8E-5). This result indicated that D254Mfs^*^68 retained partial function, although protein expression was undetectable. Transfectants with W43R, R161Q, R225X and R275X showed radiosensitivity, which appeared close to that of wild-type XRCC4 transfectant, indicating that these mutants were mostly functional when overexpressed. Nevertheless, W43R (p = 0.042 with wild-type), R161Q (p = 0.018 with wild-type), R225X (p = 0.018 with wild-type) and R275X (p = 0.014 with wild-type) transfectants showed slightly but significantly higher sensitivity than wild-type XRCC4 transfectant, indicating that these mutants were not fully functional.

### Nuclear localization of XRCC4 mimicking mutations in patients

**Fig. 4.**
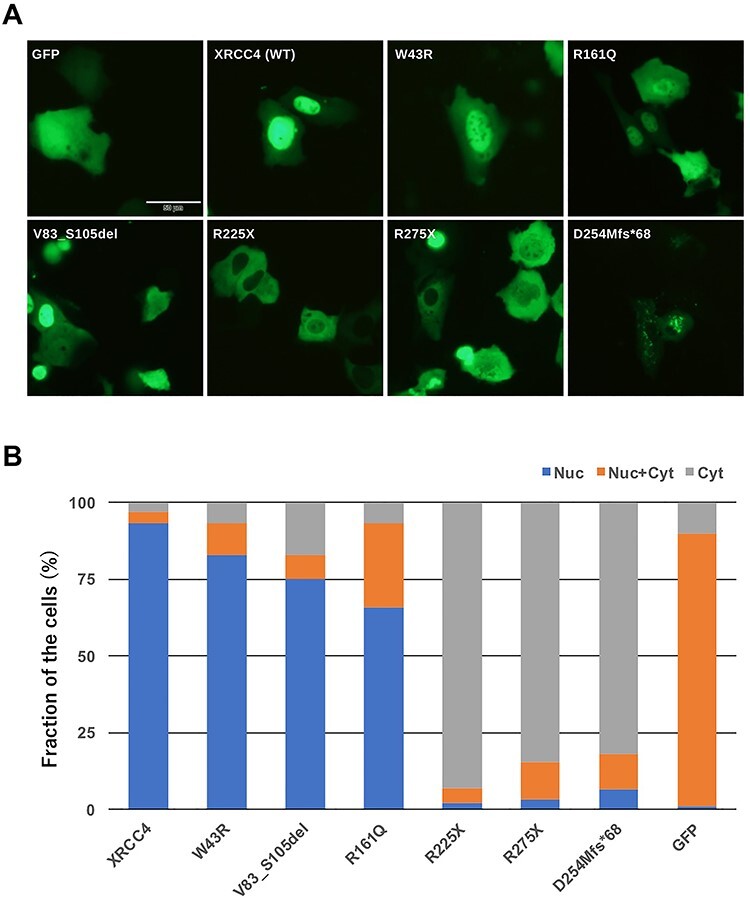
Subcellular localization of wild-type and disease-associated mutants of XRCC4 tagged with GFP. A, images of fluorescent microscopy. B, quantification of microscopic observations. Cells showing nuclear distribution (Nuc), nuclear plus cytoplasmic distribution (Nuc + Cyt) and cytoplasmic distribution (Cyt) were counted. Total number of counted cells was 100–128 for each transfectant.

To evaluate the ability of disease-associated mutants of XRCC4 to localize to the nucleus, cDNA for GFP-XRCC4 were generated and transiently expressed in human osteosarcoma U2OS cells. V83_S105del, W43R, R161Q, as well as wild-type XRCC4 localized to the nucleus. On the other hand, R225X, R275X and D254Mfs^*^68 localized to the cytoplasm rather than to the nucleus (Fig. 4). These observations agreed with an earlier study demonstrating that putative nuclear localization signal including Lys271 was essential for the nuclear localization of XRCC4 [15]. It may be noted that D254Mfs^*^68 showed punctated localization in the cytoplasm, which was distinct from the localization of R225X and R275X.

## DISCUSSION

As several patients of microcephaly and growth defect, with no overt immunodeficiency, were reported to bear mutations on the XRCC4 gene, we evaluated their functionality in terms of their ability to correct radiosensitivity of XRCC4-deficient M10 cells, along with protein expression and nuclear localizing ability. Results showed varying degrees of functionality of disease-associated XRCC4 mutations.

Among the mutants, V83_S105del transfectant showed the highest radiosensitivity. A study by de Bruin *et al.* demonstrated defective blunt end joining of plasmid in fibroblast derived from the patients harboring homozygous mutation [43]. V83_S105del was expressed at a similar level to wild-type XRCC4 in M10-transfectant and could be localized to the nucleus in U2OS. Additionally, LIG4 expression in V83_S105del was comparable to that in wild-type XRCC4 transfectant. However, this mutant is presumed to be defective in interaction with XLF, which requires XRCC4 amino acids 59–106 [17, 52]. As the radiosensitivity of V83_S105del transfectant was not significantly different from that of the control CMV vector transfectant, it is not excluded presently that this mutant is null functional. Nevertheless, this mutation found in the patients was thought to affect splicing [43], so there is a possibility of the expression of normally spliced transcript even at a low abundance, although this has not been found in the patients.

D254Mfs^*^68 transfectant showed the second highest radiosensitivity. The expression of D254Mfs^*^68 was low and initially undetectable in M10-transformant, suggesting its low stability. This is agreeable with the study by Guo *et al.* showing that D254Mfs^*^68 was unstable due to proteasomal degradation [45]. They also showed that when D254Mfs^*^68 or XRCC4(1–253) was overexpressed in the fibroblast from patient (P11 in Table 1), DSB repair ability measured by γ-H2AX foci was almost fully restored. Therefore, DSB repair defects of D254Mfs^*^68 mutant might be mainly due to low abundance. In this study, D254Mfs^*^68 was expressed at similar levels to wild-type XRCC4. The LIG4 expression in the D254Mfs^*^68 transfectant was comparable to the wild-type XRCC4 transfectant, but the D254Mfs^*^68 transfectant was more radiosensitive than the wild-type XRCC4 transfectant. Additionally, punctated cytoplasmic localization of D254Mfs^*^68 was observed. Guo *et al.* also reported that XRCC4 proteins, i.e. R225X and D254Mfs^*^68, distributed in the cytoplasm as well as in the nucleus of the fibroblast from patient (P11 in Table 1) [45]. The immunofluorescent image in their study showed punctated subnuclear localization of XRCC4 in the nucleus. Considering this, the attachment of aberrant sequence due to frameshift in D254Mfs^*^68 might have driven peculiar distribution in the nucleus and in the cytoplasm.

R225X was expressed at a similar level to wild-type XRCC4 and W43R, R275X and R161Q were expressed at higher levels than wild-type XRCC4 in M10-transfectants. They could correct the radiosensitivity of M10 to the extent close to wild-type XRCC4. LIG4 was expressed at a comparable level in the transfectants of these mutants and the wild-type XRCC4 transfectant. These results indicated that these mutants were mostly functional when expressed at a sufficient level and, therefore, XRCC4 defects in patients might be primarily due to the low abundance of the proteins. Nonetheless, the radiosensitivity of the transfectants of these mutants was slightly, but significantly higher than that of wild-type XRCC4 transfectant, suggesting that these mutants were not fully functional.

In the study by Murray *et al.*, the XRCC4 protein was undetectable in fibroblasts from a patient with homozygous W43R mutation (P2 in Table 1) [41]. This patient’s fibroblast also showed substantial increase in radiosensitivity and reduction in DSB repair [41]. Guo *et al.* showed that exogenous expression of W43R mutant in patient P11 only partially restored DSB repair ability measured by γ-H2AX foci counting, although W43R was expressed at a similar level to wild-type XRCC4 [45]. Trp43 is highly conserved among XRCC4 homologues, and in XLF and PAXX in a wide range of eukaryotic species, suggesting its role in maintaining the structure of globular head domains of these molecules [7, 8].

In the study by Bee *et al.*, XRCC4 protein expression was undetectable in patient with homozygous R225X mutation (P7–1 and P7–2 in Table 1) [42]. Profound decrease of XRCC4 mRNA in these patients’ fibroblasts suggested the involvement of nonsense-mediated decay due to longer C-terminal untranslated region than wild-type XRCC4 mRNA [42]. Although R275X mutation was reported in four patients (P4–1, P-4-2, P5 and P10 in Table 1), protein expression level was not known [41, 44]. R225X and R275X also showed cytoplasmic localization in this study. The present results might suggest the possible importance for DSB repair function of putative nuclear localization signal and/or other parts, including potential post-translational modification sites, which are missing in these mutants (Fig. 1).

Arg161 is involved in a nonsense mutation R161X (P3 in Table 1) [41] and a missense mutation R161Q (P9–1, P9–2 and P9–3 in Table 1) [44]. Nonsense mutation R161X was not analyzed in this study, as it is expected to lack interaction with LIG4. The mutation c.G482A, which caused R161Q, also produced two alternatively spliced products which potentially encode XRCC4 with F106Ifs^*^1 and V47Dfs^*^5 mutations, respectively [44]. RT-PCR analysis showed that mRNA encoding F106Ifs^*^1 was the most abundant among the three splicing products, with a great decrease in mRNA encoding R161Q, in the fibroblast from P9–1 [44]. As a result, XRCC4 protein expression in fibroblast from P9–1 was greatly reduced as compared to normal fibroblast. Although Arg161 is not as highly conserved as Trp43, it is located between the dimerization domain and LIG4-binding domain, and also included in the interacting region with Intermediate filament family orphan 1 (IFFO1), probably contacting with Gln515 in IFFO1 [53].

In summary, the defects of XRCC4 in disease patients harboring these mutations might be due to insufficiency in protein quantity and impaired functionality. The present study in conjunction with earlier studies underscores the importance of XRCC4’s DSB repair function in normal development. On the other hand, these patients showed normal immunological functions. Disease-associated XRCC4 mutations, except for V83_S105del, were partially, but not null, functional. The possibility of V83_S105del being null functional could not be excluded here, but there remains a possibility of the expression of normally spliced transcript even at a low abundance. Other mutations H9Tfs^*^8 and R161X, which were not analyzed in this study, can be null functional, but they are carried by patients with R275X and R225X in the other allele, respectively. Considering this, all the patients with XRCC4 mutations identified to date may have retained low but partial function of XRCC4. Although the role of XRCC4 in V(D)J recombination is established mainly in rodent cells [2, 37, 38], a recent study showed that human XRCC4-knockout cell, generated from colon cancer HCT116 cell, was defective in V(D)J recombination [54]. It was shown that minimal level of DNA-PK activity was sufficient for V(D)J recombination, whereas higher activity was required for radioresistance [55]. Likewise, the requirement for XRCC4 might be different quantitatively and/or qualitatively between the repair of radiation-induced DSB and V(D)J recombination.

## Supplementary Material

SupplementaryFigureS1_rrab016

SupplementaryFigureS2_rrab016

SupplementaryTable1_rrab016

## PRESENTATION AT CONFERENCE

A part of this work was presented in the Japan Radiation Research Society 59^th^ and 60^th^ Annual Meetings, Radiation Research Society 62^nd^ and 63^rd^ Annual Meetings, 33^rd^ International Symposium of Radiation Biology Centre, Kyoto University and International Congress of Radiation Research 2019.
