# Supplementary figures and images for: Functional analysis of XRCC4 mutations in reported microcephaly and growth defect patients in terms of radiosensitivity
Source: J Radiat Res. 2021 Apr 12;62(3):380–9. doi: 10.1093/jrr/rrab016 (PMC8127669; doi:10.1093/jrr/rrab016)

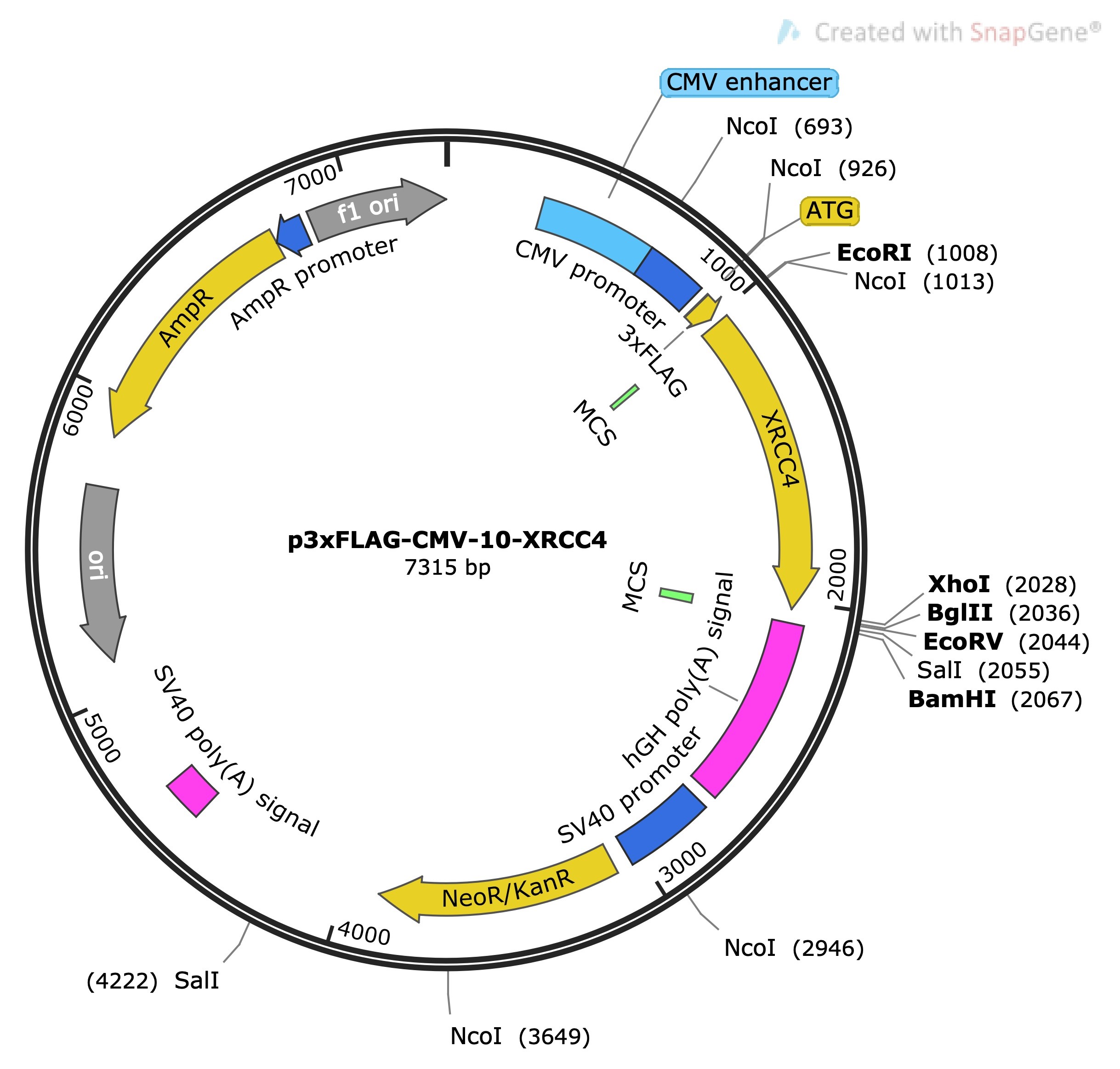

Supplement: SupplementaryFigureS1_rrab016 [file supplementaryfigures1_rrab016.jpeg]

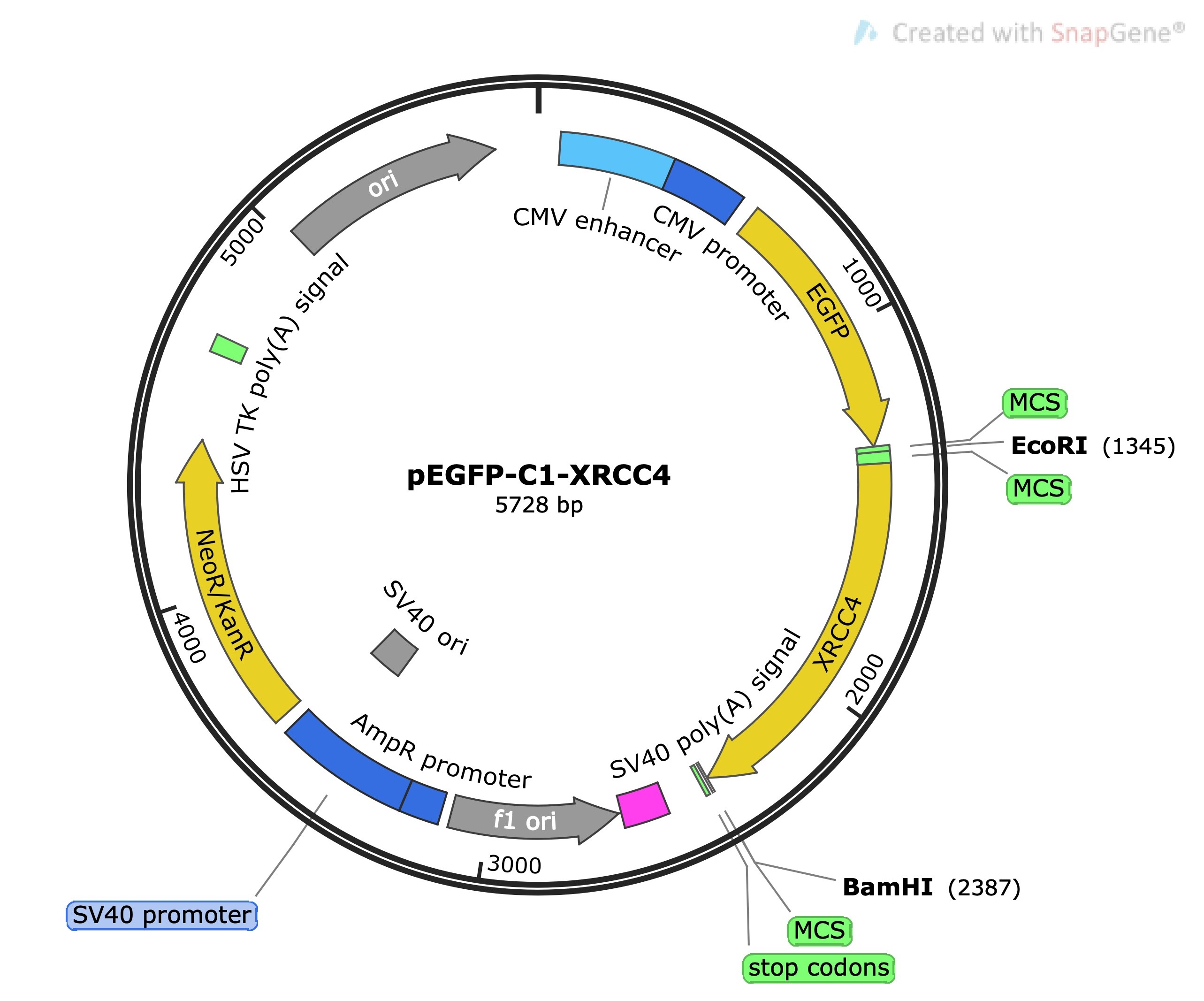

Supplement: SupplementaryFigureS2_rrab016 [file supplementaryfigures2_rrab016.jpeg]
