# Supplementary material for: Functional analysis of XRCC4 mutations in reported microcephaly and growth defect patients in terms of radiosensitivity
Source: J Radiat Res. 2021 Apr 12;62(3):380–9. doi: 10.1093/jrr/rrab016 (PMC8127669; doi:10.1093/jrr/rrab016)
Supplement: SupplementaryTable1_rrab016 [file supplementarytable1_rrab016.docx]

**Table S1. Profile of Patients associated with XRCC4 Mutations**

| Patient | Gender | Country of origin | Consan-guinuity of parents | Change in nucleotide sequence | Change in amino acid sequence | Clinical features | | | Reference |
| --- | --- | --- | --- | --- | --- | --- | --- | --- | --- |
|  |  |  |  |  |  | Microcephaly  (OFC) | Short statue  (Length/height) | Other features |  |
| P1 | F | Saudi Arabia | Y | c.T127C  (Homozygous) | p.W43R | Y  (4y:-8.3SD) | Y  (4y:-7.1SD) | Speech delay, triangular bird-like face, short philtrum | [40] |
| P2 | M | Saudi Arabia | NR | c.T127C  (Homozygous) | p.W43R | Y  (Birth:-4.87SD;  3y1m:-8.3SD) | Y  (Birth:-2.52SD;  3y1m:-4.7SD) | NR | [41] |
| P3 | M | Morocco | NR | c.C481T  c.C673T | p.R161X  p.R225X | Y  (Birth:-4.57SD;  2y9m:-8.3SD) | Y  (Birth:-6.32SD;  2y9m:-5.7SD) | Gastrostomy, ectopic kidney, small bilateral kidneys, chronic lung disease | [41] |
| P4-1# | M | Italy | NR | c.C25del  c.C823T | p.H9Tfs*8  p.R275X | Y  (Birth:-2.9SD  8y4m:-5.6SD) | Y  (Birth:-4.49SD  8y4m:-2.4SD) | NR | [41] |
| P4-2# | M | Italy | NR | c.C25del  c.C823T | p.H9Tfs*8  p.R275X | Y  (Birth:-1.83SD;  4y:-8.0SD) | Y  (Birth:-5.38SD;  4y:-4.5SD) | NR | [41] |
| P5 | M | France | NR | c.C25del  c.C823T | p.H9Tfs*8  p.R275X | Y  (Birth:-4.28SD;  9y:-5.8SD) | Y  (Birth:-2.71SD;  9y, -1.8SD*) | Unilateral renal agenesis, cryptorchidism | [41] |
| P6 | M | United Kingdom | NR | c.C25del  c.G-10-1T | p.H9Tfs*8  splicing defect | Y  (Birth:-2.9SD;  5m, -8.9SD) | Short statue (Birth:-6.56SD;  5m, -7.2SD) | Eczema | [41] |
| P7-1# | M | Italy | Y | c.C673T  (Homozygous) | p.R225X | NR | Y | Adult-onset cardiomyopathy, neurological disorders, short limbs, pes avus, bilateral cryptorchidism, hypotelorism | [42] |
| P7-2# | M | Italy | Y | c.C673T  (Homozygous) | p.R225X | NR | Y | Adult-onset cardiomyopathy, neurological disorders, short limbs, pes avus, bilateral cryptorchidism, hypotelorism | [42] |
| P8-1# | M | Chile | NR | c.T246G, c.247_315del  (Homozygous) | p.D82E, p.V83-S105del | Y  (39.9y:-3.3SD) | Y  (Birth:-2.8SD;  39.9y:-6.8SD) | Primary gonadal failure, type 2 diabetes, dyslipidemia, acanthosis, clinodactyly, cataracts, multinodular goiter | [43] |
| P8-2# | F | Chile | NR | c.T246G, c.247_315del  (Homozygous) | p.D82E, p.V83-S105del | Y  (36y:-2.9SD) | Y  (Birth:-2.3SD;  36y:-4.0SD) | Primary gonadal failure, type 2 diabetes, dyslipidemia, acanthosis, clinodactyly, anemia, gastrointestinal stromal tumor (jejunum) | [43] |
| P9-1# | M | Turkey | Y | c.G482A  (Homozygous) | p.R161Q | Y  (Birth:<-3SD;  14y:-7.5SD) | Y  (Birth:-2 SD;  14y:-5SD) | Mild intellectual disability, facial Dysmorphism | [44] |
| P9-2# | M | Turkey | Y | c.G482A  (Homozygous) | p.R161Q | Y  (Birth:<-3SD;  10.5y:-6.5SD) | Y  (Birth:NR;  10.5y:-5SD) | Mild intellectual disability, facial Dysmorphism | [44] |
| P9-3# | M | Turkey | Y | c.G482A  (Homozygous) | p.R161Q | Y  (Birth:<-3SD;  6m:-4.5SD) | Y  (Birth:NR;  6m:-3SD) | Mild intellectual disability, facial Dysmorphism | [44] |
| P10 | F | Switzerland | NR | c.C25del  c.C823T | p.H9Tfs*8  p.R275X | Y  (Birth:-3.7SD;  14y10m:-5.0SD) | Y  (Birth:-2.8SD;  14y10m:-2.6SD) | Facial dysmorphism | [44] |
| P11 | F | United Kingdom | N | c.C673T  c.G760del | p.R225X  p.D254Mfs*68 | Y | Y | Progressive ataxia, hyperopia, diabetes mellitus, hypothyroidism, thalamic glioma, moderate hearing loss, slurred speech | [45] |

#: siblings. *Growth hormone-treated.

Abbreviations: F, female; M, male; Y, yes; N, no; NR, not reported; y, year; m, month; SD, standard deviation; OFC, occipitofrontal circumference.
